# Special Issue “Emerging Viruses 2020: Surveillance, Prevention, Evolution and Control”

**DOI:** 10.3390/v13020251

**Published:** 2021-02-06

**Authors:** Fabrício Souza Campos, Luciana Barros de Arruda, Flávio Guimaraes da Fonseca

**Affiliations:** 1 Laboratório de Bioinformática & Biotecnologia, Campus de Gurupi, Universidade Federal do Tocantins, Gurupi 77402-970, TO, Brazil; 2 Departamento de Virologia, Instituto de Microbiologia Paulo de Góes, Universidade Federal do Rio de Janeiro, Rio de Janeiro 21941-902, RJ, Brazil; 3 Laboratório de Virologia Básica e Aplicada, Instituto de Ciências Biológicas, Departamento de Microbiologia, Universidade Federal de Minas Gerais, Belo Horizonte 31270-901, MG, Brazil

This Special Issue of *Viruses* is a collection of the current knowledge on a broad range of emerging human, animal, and plant viral diseases. Emerging and re-emerging viruses represent constant and historical challenges to medical and scientific communities, being some of the major concerns for public health. The world is facing increasing numbers of outbreaks, epidemics, and pandemics, which are probably consequences of anthropogenic activities directly or indirectly impacting the climate, ecosystems, and biodiversity. These are intensified by local and international migration of people and product trading, allowing the rapid spread of potential pathogens. The year 2020 was profoundly marked by the rapid and severe spread of SARS-CoV2, establishing the worst pandemic of the century until now with 102.8 million cases and 2,219,066 deaths in more than 200 countries as of 31 January 2021 [1]. Indeed, the COVID-19 pandemic has been a catastrophic demonstration of the impact of emerging viruses in public health systems and on the global economy, reinforcing the importance of virus research and surveillance. In this Issue, we are proud to have received a total of 55 manuscript submissions with an acceptance rate of 41.82% (23 papers) from colleagues from different parts of the world working on a wide range of different viruses. Original manuscripts and reviews related to the surveillance, detection, and evolution of emerging viruses—from local outbreaks to worldwide viral pandemics— were received, including the description of new methods and the adaption or creation of technologies for the study, prevention, and control of emerging viruses.

In the face of the current pandemic, many published manuscripts were related to SARS-CoV-2. Klein et al. [2] presented a magnetic bead-based protocol for SARS-CoV-2 RNA extraction that used less expensive devices but yielded RNA extracts comparable to the commercially available QIAcube viral RNA extraction kit, providing similar sensitivity and specificity by the commonly applied detection methods RT-qPCR and reverse transcription loop-mediated isothermal amplification (RT-LAMP), using E and N targets, respectively. Stoddard et al. [3] described an ensemble molecular docking approach and proposed a designed guideline to virtually trial potential drug candidates to the SARS-CoV-2 main protease. Liu et al. [4] reviewed several aspects of antiviral immune response and evasion mechanisms presented by coronaviruses that have caused epidemic diseases in humans and domestic animals. The discussed mechanisms included the hijack of antigen-presenting cells to virus dissemination through mucosal tissues; escape from RNA sensors by shielding RNA intermediates in replication organelles and 2′-O-methylation modification of virus RNA; antagonization of interferon-mediated signal transduction, and inhibition of interferon stimulated genes (ISG). Pirnay et al. [5] characterized a SARS-CoV-2 outbreak in a Belgian military education and training center in Maradi, Niger. Viral load could be detected in symptomatic and asymptomatic individuals, and analysis of the genome sequences suggested that the soldiers and trainers were infected in Africa. The study indicated the importance of viral surveillance and contributed to the implementation of virus and antibody testing in military personnel before and after missions abroad. Khaiboullina et al. [6] evaluated whether UV-induced photocatalytic properties of nanosized TiO2 (TNPs) would function as a virus deactivation tool using glass coverslips and HCoV-NL63 as a coronavirus model. Finally, Fakhroo et al. [7] reviewed the correlation between several viral, host, and environmental factors with the susceptibility to SARS-CoV-2 infection and disease severity. Viral variants, individual comorbidities, genetics, microbiome, and blood group, as well as different medications, metabolome, vitamins, and immune status were discussed, indicating a combination of elements that may be related to potential high-risk groups for COVID-19.

Interestingly, other articles discussing diversity, transmission, and pathogenesis of human and animal infection by bat-borne pathogens, besides coronaviruses, were also published in this Issue. Soman Pillai et al. [8] reviewed several characteristics of recent outbreaks of Nipah virus occurring in Malaysia, Bangladesh, and India, discussing the clinical features, virus strains, diagnosis, treatment, and socio and environmental factors that may have contributed to the outbreaks. Those descriptions may increment the current knowledge regarding viral dynamics and may help in surveillance and control measurements.

Other potential zoonotic viruses include the avian influenza virus (AIV) and the complex overlap in waterfowl migratory pathways, which has helped establish numerous occurrences of genetic reassortment and intercontinental AIV spread. Nguyen et al. [9] isolated a novel avian influenza H6N5 (K6) subtype from a fecal sample of wild bird, collected during annual surveillance in South Korea in 2018. Genomic characterization was performed, indicating the K6 virus to be of North American origin, with partial homology to an H6N5 strain. The authors also evaluated in vitro and in vivo replication in mammalian systems, suggesting potential adaptability and host jumping of new isolates, reinforcing the importance of constant surveillance.

Arboviruses such as Dengue virus, Japanese encephalitis virus, West Nile virus, Chikungunya virus (CHIKV), Yellow fever (YF) and Zika virus (ZIKV), also have been frequently associated with outbreaks and epidemics, causing diseases with high morbidity and mortality rates in developed and undeveloped countries, sometimes under a deficient surveillance program. Diagnosis protocols were discussed in two articles here. Almeida et al. [10] evaluated the influence of mosquito tissue, RNA extraction, and cDNA synthesis for the accuracy of ZIKV detection by RT-dPCR. Bagno et al. [11] compared the performance of IgG and IgM ELISA tests to CHIKV, using two CHIKV-E2 recombinant antigens produced in prokaryotic and eukaryotic expression systems. Although both antigens showed similar efficiency in the IgG ELISA, the eukaryotic-produced glycosylated one presented higher sensitivity and specificity in the IgM assay. Arbovirus epidemiology was also debated in this Issue. Historical aspects of YFV infection in Brazil were discussed by Oliveira Figueiredo [12], emphasizing important issues that could be learned during the recent outbreak in the country, which led to a relevant number of deaths and affected regions that were considered free of the disease. Siqueira et al. [13] identified and followed up six patients with Zika/dengue coinfection in Brazil in a Brazilian cohort. The patients did not show any warning signs or neurological clinical manifestation, with no need for hospitalization. Thus, the authors suggest that a continuous specific laboratory confirmation for both dengue and Zika viruses should be part of surveillance systems to avoid a late detection of ZIKV circulation. Oropouche orthobunyavirus (OROV) and Mayaro virus are two emerging viruses that have been reported as candidates for the next big epidemics in countries from South America, such as Brazil. Ribeiro Amorim et al. [14] evaluated OROV capacity to infect and persist in human lineages of leukocytes and peripheral blood mononuclear cells (PBMC), and evaluated the pattern of the elicited innate immune response after different time points post infection.

Many viruses are involved in the development of cancer. Epstein Barr virus (EBV) is a ubiquitous oncogenic virus associated with nasopharyngeal cancer (NPC), and different strains have been associated with the disease in distinct countries. Ayee et al. [15] detected and characterized EBV in patients diagnosed with NPC in Ghana and controls. The study indicated that EBV genotype 2 was predominant in NPC patients with higher viral load, whereas genotype 1 was predominant in control individuals, suggesting that the detection and quantification of EBV load can be used as a noninvasive biomarker for the diagnosis of NPC.

Human pathogens can also be transmitted through water. With this in mind, Lartey et al. [16] analyzed more than 1000 samples of diarrheic stool collected from children <5 years from 2008 to 2017 and found different norovirus genotypes, showing that the severity of clinical illness in children infected with GII.4 norovirus strains was similar to illness caused by non-GII.4 strains.

Many articles discussed the epidemiology and pathogeneses of animal viruses. Cano et al. [17] reported the first isolation of a fish nidovirus from a consignment of goldfish at the United Kingdom border. The article described the full genome analysis and phylogeny of the virus, in vitro replication in different fish cell lines, and susceptibility of goldfish and common carp to in vivo experimental infection, with the record of clinical signs. This study reinforces the potential risk of novel and emerging pathogens being introduced to recipient countries via the international ornamental fish trade and the importance of regular full-health screens at border inspection posts to reduce this risk.

Pathogens affecting livestock and domestic animals represent an important problem in the current one-health view and approaches in relation to infectious diseases. Startled by the detection of African swine fever virus (ASFV) on wild boars in Belgian locations close to the French border, Bonnet et al. [18] presented a review paper on the possible role of arthropods in the dissemination of ASFV—a pathogen with severe implications for the pig farming industry, including significant sanitary and economic impact. In this review, authors discussed how arthropods could facilitate the dissemination of ASFV through France as a vector-borne disease (VBD). Examples of arthropod groups that could work as putative vectors for ASFV transmission were scrutinized in detail, including their behavior, biological properties and eventual data that implicate them in possible ASFV transmission events. The authors concluded that there are important gaps in the current knowledge of vector biology and understanding of how these vectors could be implicated in ASFV spread. 

In another review, Li and Zheng et al. [19] discussed the host response triggered by infectious bursal disease (IBD), which is an acute immunosuppressive avian disease, affecting mostly chickens. The authors focused on the role micro RNAs previously characterized in chickens and their mammalian counterparts, which expression affects not only viral replication, and also cell apoptosis and innate immune-response modulation.

Keeping the one-health approach in mind, the next article in the Special Issue focused on yet another virus family capable of infecting a wide range of vertebrate hosts: the circoviruses. Circoviruses, which are small, circular, single-stranded DNA-bearing viruses, have been identified in penguins, and a penguin circovirus (PenCV) has been described in the past. In their article, Levy et al. [20] visited seven Antarctic breeding colonies in different regions of Antarctica and collected 75 cloacal swab samples from adults and chicks of three species of penguins. Samples were screened for PenCV DNA, and two new genetic variants were identified: one in an Adélie Penguin (*Pygoscelis adeliae*) and another in a Chinstrap Penguin (*Pygoscelis antarcticus*). The circovirus sequences reported in this manuscript were approximately 12% divergent to those previously reported. Interestingly, the sequences reported here were more similar to each other than to the previously reported genomes, and the fact that they came from closely located colonies may indicate the intense circulation of penguin circoviruses in the region.

Han et al. [21] identified nine new husavirus strains in China using metagenomic approaches. These new genomic sequences were found in stool samples from healthy children. Husavirus strains belong to the order *Picornavirales* and appear to have a wide geographic distribution, but their specific hosts as well as other relevant virological information are largely unknown. The authors suggest, based on their analysis, that these posalike viruses may represent a new virus family within the order *Pircornavirales*, and they may have come from parasites infecting the guts of mammals and other animals. Also regarding viruses from invertebrates, in the next article Hooper et al. [22] investigated a mass mortality event of giant freshwater prawn (*Macrobrachium rosenbergii*) larvae in Bangladesh. Using metatranscriptomic sequencing, the authors identified a new virus species within the *Nidovirales* order, baptized as Macrobrachium rosenbergii golda virus. Although circumstantial, the link between the found virus’s genomes and larvae mortality was suggested on the basis of genomic similarity to other viruses causing mass mortality of prawn and the consecutive finding of this virus RNA in material coming from distinct mortality events and farms. 

Returning to viruses from vertebrates, in the next paper Abade dos Santos et al. [23] demonstrated that recombinant myxoma virus (MYXV) can effectively infect and cause myxomatosis in wild and domestic rabbits. The recombinant MYXV—an MYXV strain that encompasses a 2.8Kb insert of duplicated genes on its genome—was thought to occur predominantly in hares, but the finding that the virus causes mortality in rabbits raises concerns about conservation of wild rabbits from Iberian Europe and indicates that monitoring programs should be implemented in order to secure wildlife welfare.

Finally, Slhessarenko et al. [24] published the 30th meeting of the Brazilian Society for Virology (SBV), which was held for the first time in Central Western Brazil, in 2019. The meeting attendants included researchers and students from all the Brazilian regions and abroad, who presented a great variety of recent unpublished studies on environmental, basic, animal, human, plant, and invertebrate virology. Although this Special Issue was prepared by *Viruses* in collaboration with the Brazilian Society for Virology, we are very grateful for having received submissions from several countries and hundreds of colleagues. We believe that the success of this Special Issue is the consequence of the great job done by the *Viruses* staff and all authors who decided to submit their research here. On behalf of the Brazilian Society of Virology, thank you!
